# Interactive effects of cooking method, genotype and environment on nutritional and antinutritional quality of quinoa (*Chenopodium quinoa* Willd.) seeds

**DOI:** 10.1002/jsfa.71022

**Published:** 2026-08-28

**Authors:** Rahma Goussi, Rim Ben Youssef, Hamza Gadhoumi, Hatem Ben Jouira, Arafet Manaa

**Affiliations:** ^1^ Laboratory of Extremophile Plants, Centre of Biotechnology of Borj Cedria Hammam‐Lif Tunisia

**Keywords:** quinoa seeds, cooking, boiling, steaming, nutritional properties, genotype × environment interaction

## Abstract

**BACKGROUND:**

Quinoa has gained global attention as a climate‐resilient crop with high nutritional value and strong potential to enhance food security. Quinoa seeds are characterized by high‐quality protein with a balanced essential amino acid profile and are rich in dietary fiber, vitamins, essential minerals, healthy lipids and bioactive compounds. This study aimed to evaluate the effects of boiling and steaming on the nutritional and antinutritional properties of five quinoa genotypes grown in two contrasting regions.

**RESULTS:**

Both environmental and genetic factors significantly affected key agronomic traits, with clear genotype × environment interactions. Overall, Q3 exhibited the best nutritional profile across both regions, combining high protein, starch, vitamin E, antioxidant capacity and the lowest levels of antinutritional compounds (saponins, tannins and phytic acid). By contrast, Q5 showed the least favorable profile, characterized by low yield, high saponin and tannin contents, and less consistent nutritional improvements after processing. Steaming consistently preserved or enhanced protein content, soluble sugars, polyphenols, flavonoids and DPPH (i.e. 2,2‐diphenyl‐1‐picrylhydrazyl) radical scavenging activity across all genotypes and sites, with the greatest protein increases observed in genotypes Q4 and Q5 from the northern site. By contrast, boiling significantly reduced protein, soluble sugar, starch, polyphenol and flavonoid contents in most genotypes. Both boiling and steaming significantly reduced phytic acid content at the same time as inducing genotype‐ and site‐dependent changes in saponin and tannin levels.

**CONCLUSION:**

These results demonstrate the strong genotype‐dependent effects of thermal processing and provide new insights into the interactions between genotype, environment and cooking method in shaping the nutritional composition of quinoa seeds. © 2026 The Author(s). *Journal of the Science of Food and Agriculture* published by John Wiley & Sons Ltd on behalf of Society of Chemical Industry.

## INTRODUCTION

Climate change is increasing global food insecurity by disrupting temperature regimes, precipitation patterns, and the frequency and severity of extreme weather events, thereby directly undermining crop growth, yield stability and production reliability.^1^ These climate‐driven stresses are projected to substantially reduce global agricultural productivity, particularly in vulnerable regions. Major staple crops such as wheat, maize and rice, which collectively supply a substantial share of global caloric intake, are expected to experience considerable yield declines under future climate scenarios.^2^, ^3^ Consequently, the capacity of current cropping systems to sustain the food supply for a rapidly growing population is becoming increasingly uncertain. With the global population projected to reach 9.8 billion by 2050, food demand is expected to rise sharply, placing unprecedented pressure on already constrained agricultural systems. To meet this demand, global cereal production will need to increase by approximately 70–100% by 2050 to adequately feed the projected population.^4^ Addressing this challenge will require the development of agricultural systems that not only enhance productivity, but also promote environmental sustainability and social equity.^5^ In this context, quinoa (*Chenopodium quinoa* Willd.) has emerged as a promising alternative crop for enhancing global food security, particularly following the declaration of 2013 as the International Year of Quinoa by the United Nations (Food and Agriculture Organization). Compared with traditional cereal crops such as wheat, maize and rice, quinoa exhibits superior resilience to adverse conditions.^6^ As a facultative halophyte belonging to the Amaranthaceae family, a taxon renowned for its high proportion of salt‐tolerant species, quinoa is highly adaptable to a broad range of agro‐ecological environments.^7^ Its exceptional tolerance to multiple abiotic stresses, including salinity,^8^, ^9^ drought,^10^, ^11^ frost^12^ and heat,^13^ makes it exceptionally well suited for cultivation in marginal soils.

Native to Chile and Peru, quinoa served as a staple food for the Inca Empire in the Andean region for over 5000 years. Considered one of the most nutritious seeds in the world, quinoa is a rich source of nutrients, with protein content ranging from 10% to 24% depending on the genotype. These proteins are of high quality, providing a balanced profile of essential amino acids, including lysine, tryptophan and cysteine.^14^ The seeds also contain 6–11% oil, characterized by a high proportion of polyunsaturated fatty acids and a favorable ω‐6/ω‐3 ratio. In addition, quinoa is rich in dietary fiber, vitamin E and essential minerals such as magnesium, iron, manganese, copper, calcium and phosphorus. Quinoa also contains bioactive compounds with high antioxidant activity, including polyphenols, carotenoids and tocopherols. As a naturally gluten‐free pseudo‐cereal with a low glycemic index, quinoa has been shown to reduce the risk of metabolic diseases, benefiting populations such as children, individuals with celiac disease and people with diabetes.^15^ Notably, these nutritional properties are influenced by genotype, cultivation location, soil characteristics and fertilization practices, as well as by post‐harvest processes, including cleaning, saponin removal, storage and cooking methods.^16^

The most common food cooking methods, boiling and steaming, are widely used because of their simplicity and convenience, and they influence nutrient bioavailability, nutritional value and digestibility.^17^ Variations in nutritional properties, including their stability, loss or enhancement, following cooking processes are largely dependent on the food type, processing method and the characteristics of specific nutrients.^18^ Previous studies have demonstrated that thermal processing can lead to a decrease in phenolic compounds, thereby reducing the antioxidant capacity of mulberry leaves.^19^ By contrast, several studies have reported that boiling can improve protein digestibility by inducing protein denaturation at the same time as retaining nutrients in lentils.^20^ Both boiling and steaming have also been reported to increase dietary fiber content in whole grains, including barley, buckwheat, quinoa, sorghum and oats.^21^, ^22^ Moreover, both steaming and boiling have been found to enhance phenolic and tannin content in wheat.^23^ To our knowledge, few studies have directly compared the effects of steaming and boiling on quinoa seed quality. Evaluating the effects of cooking methods is therefore essential to better understand their impact on nutritional quality and to guide consumers in selecting cooking practices that maximize health benefits. Accordingly, this study aimed to evaluate the effects of boiling and steaming on the nutritional and antinutritional properties of five quinoa genotypes grown in two contrasting regions. Specifically, we investigated how genotype, growing environment and cooking methods interact to influence agronomic performance, seed compositions, including protein, sugars, starch, vitamin E, antioxidants (e.g. phenolic compounds and flavonoids) and antinutritional factors (saponins, phytic acid and tannins), providing insight into the factors shaping quinoa seed quality.

## MATERIALS AND METHODS

### Quinoa seed collection

The plant material used in this study included 06 genotypes of quinoa (*Chenopodium quinoa* Willd.) and their characteristics and features are given in the Table 1. These genotypes were selected based on a preliminary screening performed in our laboratory. Seeds of these genotypes were collected in 2024 from two contrasting regions in Tunisia. The first demosite was located in the southern Tunisian Desert (Douz, Noueil region, 36°37′14.0″N 10°32′42.4″E). This location is known for being one of the driest regions in Tunisia.^24^ The second demosite was located in the northern Tunisia (Grombalia region, 33°30′25.2″N 8°50′11.2″E) considered as a humid region. The geographic distribution, climatic conditions and soil characteristics of these two demosites are presented in the Supporting information (Table S1).

**Table 1:** Characteristics of quinoa genotypes tested

	ID	Genotypes	Accession	Seed origin	Seed source	Seed color	Vegetative cycle (days)
1	Q1	Regalona		Chile	Chile	White	100–120
2	Q2	QQ57	PI 614885	Biobío, Chile	USDA	White	130–140
3	Q3	Faro	PI 634920	Chile	USDA	White	150–160
4	Q4 (N)	C0‐407/	PI 698742	USA	USDA	Pink	130–140
5	Q4 (S)	Amarilla sacaca		Peru	ITDAS	Orange	170–180
6	Q5	QQ654	PI 634921	Chile	USDA	Pink	130–140

### Seed sampling, preparation and cooking process

Quinoa panicles were manually collected from the two demosites on February 2024 from the northern demosite and on June 2024 from the south and then purified from contaminant and dried at air temperature. Quinoa seeds were manually isolated from panicles and divided into three pools for later analysis: (RS) raw seeds. (BS) boiled seeds: seeds were transferred to a beaker at a ratio of 100 g of quinoa to 300 mL of deionized water. The water was not preheated; the seeds were directly immersed in deionized water at room temperature (22–25 °C) and heated from the start. The total cooking time was 20 min, including the heating period from room temperature to boiling, and continued until complete water evaporation. (SS) steamed seeds: the seeds were placed in stainless steel pots and steamed over boiling water on a hot plate for 45 min. Steaming was continued until the seeds became fully hydrated and smooth in texture. The boiling and steaming processes were each repeated three times, and no saponin removal process was applied. The obtained BS and SS were dried, then grounded in a fine powder and stored at −20 °C prior to nutritional analysis.

### Nutritional factors

#### Protein extraction and quantification

Aliquot of ground seed powder was extracted (0.5 g, 250 mL) at 4 °C in 100 mm Tris–HCl buffer (pH 8.0) containing 10 mm EDTA, 50 mm KCl, 20 mm MgCl_2_, 0.5 mm phenyl methyl sulfonyl fluoride, 1 mm dichloro diphenyl trichloroethane, 0.1% (v/v) Triton X‐100 and 10% (w/w) polyvinylpyrrolidone. The homogenate was centrifuged at 10 000 g for 15 min at 4 °C, and the supernatant was clarified by filtration. The supernatants were collected and used later for protein content analysis according to the Bradford protocol.^25^ The absorbance was measured at 595 nm using UV‐visible spectrophotometry and the protein concentration was estimated using bovine serum albumin as standard.

#### Vitamin E content

Determination of vitamin E (*α*‐tocopherol) was assayed as earlier described by Backer *et al*.^26^ Briefly, ground seeds (0.25 g) were homogenized with a mixture of petroleum ether and ethanol (2:1.6 v/v); the extract was centrifuged at 6000 g rpm for 20 min. To 1 mL of obtained supernatant, 0.2 mL of 2,2‐dipyridyl (0.5%) and 0.5 mL of FeCl_3_ (0.2%) were added, mixed thoroughly, and then kept in dark for 5 min. The resulting red product was diluted with 4 mL of distilled water and mixed well. The absorbance of the aqueous layer was then measured at 520 nm using a spectrophotometer. The vitamin E content was calculated using extrapolation on a standard curve made with a known amount of *α*‐tocopherol.

#### Soluble sugar content

Total soluble sugars were estimated using the anthrone reagent method, with d‐glucose as the standard, following Yemm and Willis.^27^ Approximately 250 mg of ground seeds were homogenized in 5 mL of 70% ethanol, vortexed for 5 min, and centrifuged at 3600 g for 10 min at 15 °C. Then, 200 μL of the supernatant was transferred into glass tubes, followed by the addition of 1 mL of anthrone reagent. Briefly, 200 μL of the samples was mixed in a glass tube with 1 mL of anthrone vortexed and incubated in water bath at 100°C for 10 min. The reaction was stopped with ice and the content was measured at 625 nm using d‐glucose as a standard curve.

#### Starch content

Starch content was determined using the iodine method, as previously described by Vainio^28^ and later modified by Bahdanovich *et al*.^29^ Briefly, 0.1 g of ground seeds was extracted in 10 mL of a mixture of 1 n HCl and 1 n KOH, followed by heating at 100 °C for 15 min. The extracts were then sonicated for 10 min, after which the supernatants were collected. An aliquot of 0.05 mL of each sample was mixed with 0.1 mL of KI/I_2_ solution and adjusted to a final volume of 5 mL with distilled water. Starch content was quantified spectrophotometrically at 720 nm using a d‐glucose standard calibration curve.

### Bioactive and antinutritional compounds

Hydro ethanolic extract for bioactive and anti‐nutritional parameters was prepared following the method of Miranda *et al*.^30^ with slight modifications. Briefly, 1 g of dried and ground RS, BS and SS was mixed with 10 mL of ethanol (70%), sonicated for 30 min at 40°C. The mixture was allowed to stand at room temperature, then filtered and the solvent was removed using a rotary evaporator, followed by lyophilization. The resulting crude extract was weighed and re‐dissolved in distilled water to a final concentration of 10 mg mL^–1^. This extract was, subsequently, used for the quantification of saponins, total polyphenols, flavonoids, and tannins.

#### Saponin quantification

Saponins quantification was assessed using the colorimetric method adapted from Patel *et al*.^31^ and Ncube *et al* ^32^ To a volume of 125 μL of extract, 125 μL of vanillin in ethanol (0.8%, w/v) and 1.25 mL of sulphuric acid in water (72%, v/v) were added. The mixture was vortexed, heated at 60 °C for 10 min, and then subjected to ice‐cooling for 3 min. Thereafter, the absorbance was measured at 520 nm using a UV‐visible spectrophotometer (Analytik Jena, Jena, Germany). Saponin content was calculated by extrapolation on a standard curve beforehand prepared with saponin standard as the tested substrate (CAS‐No 8047‐15‐2).

#### Total phenolic content

Total phenolic content (TPC) was quantified using the Folin–Ciocalteu method according to Singleton and Rossi.^33^ Briefly, 125 μL of sample was mixed with 500 μL of distilled water and 125 μL of Folin–Ciocalteu reagent (1:9, v/v). Then, 1.25 mL of sodium carbonate solution was added, and the mixture was incubated in the dark at room temperature for 90 min. Absorbance was measured at 760 nm, and TPC was determined from a gallic acid calibration curve, expressed as mg of gallic acid equivalents per gram of fresh weight (mg GAE g^−1^ FW).

#### Total flavonoid content

Total flavonoid content (TFC) was determined using the aluminum chloride colorimetric method, as described by Chang *et al*.^34^ In summary, 400 μL of plant extract was mixed with 120 μL of sodium nitrite (5%), followed by the addition of 120 μL of aluminum chloride (AlCL_3_; 10%) and 800 μL of sodium hydroxide (NaOH; 1 m). The absorbance was measured at 510 nm, and TFC was calculated from a quercetin calibration curve, expressed as mg quercetin equivalents per 100 g of sample (mg QE g^−1^ FW).

#### Tannin content

Total tannin content was measured using the vanillin‐HCl method according to Broadhurst and Jones.^35^ Briefly, 0.2 mL of seeds extract was mixed with 1 mL of vanillin reagent prepared in prepared in a mixture of HCl and acetic acid (v/v). The mixture was incubated in a water bath at 30 °C for 20 min. The absorbance was recorded at 410 nm and the tannin content was determined from a catechin (CAS: 225937‐10‐0; Sigma‐Aldrich, St Louis, MO, USA) calibration curve, and expressed as mg catechin equivalents per 100 g of extract (mg CE g^−1^ FW).

#### DPPH scavenging activity

Antioxidant activity by the 2,2‐diphenyl‐1‐picrylhydrazyl (DPPH) assay was determined by the method of Boudjou *et al*.^36^ The DPPH radical scavenging activity of the extract was determined as follows: a solution of 60 μm DPPH in methanol (MEtOH) (1950 μL) was mixed with 50 μL of each quinoa extract, vortexed and incubated at 25 °C in the dark for 60 min. Absorbance was measured at 517 nm using MEtOH as the blank. The control consisted of 50 μL of ethanol in place of the extract. Antiradical activity was expressed as the percentage inhibition of the DPPH* radical using:

$$I\%=100\times \left[1-\left(\text{absorbance of sample}/\text{absorbance of control}\right)\right]$$

#### Phytic acid content

Phytic acid content was estimated according to the procedure of Latta and Eskin^37^ with some modifications. Briefly, 500 mg of ground seed powder was extracted with 10 mL of extraction solution (10% Na_2_SO_4_ and 0.4 mol L^–1^ HCl) under sonication for 2 h. After centrifugation (6000 g for 20 min) and filtration, the extract was mixed with 1 g of NaCl. An aliquot was diluted 25‐fold, and 3 mL of the diluted extract was reacted with 1 mL of WADE reagent (3% sulfosalicylic acid supplemented with 30 μL of 60% FeCl3). The absorbance was measured at 500 nm, and phytic acid concentration was calculated from a standard calibration curve using phytic acid as the reference standard.

### Statistical analysis

The statistical analysis was performed using OriginPro 2020, SR1 (OriginLab Corp., Northampton, MA, USA). All mean ± SE values of physiological and biochemical parameters were obtained from six replicates. The means were compared using one‐way, two‐way and multivariate analysis of variance (ANOVA) followed by Duncan's multiple range tests at 95%. Additionally, principal component analysis (PCA) was conducted to explore patterns and relationships among the physiological and biochemical parameters across different factors, including genotype, environment and cooking process, to visualize the major sources of variation in the dataset.

## RESULTS

### Quinoa genotypes performance

Figure 1 illustrates the plant dry weight (DW), seed yield/plant and thousand kernel weight (TKW) of five quinoa genotypes (Q1 to Q5) cultivated at two contrasting demosites: a northern site (N), characterized by humid conditions, and a southern site (S), representing an arid environment with saline soil. Plant DW was significantly higher at the Northern site for all genotypes (Fig. 1(a)). Among them, Q2 and Q3 exhibited remarkably higher DW, indicating higher growth performance under humid conditions. Similarly, seed yield exhibited significant variation across genotypes and sites, with the northern site showing the highest seed yield (Fig. 1(b)). Moreover, the genotype Q2 and Q3 achieved the highest seed yield, whereas genotype Q5 consistently recorded the lowest yield at both sites. Despite the higher growth performance at the northern site, TKW was higher at the southern site across all genotypes, with Q1 and Q4 showing the highest values and Q5 the lowest (Fig. 1(c)). The two‐way ANOVA revealed significant effects of region, genotype, and their interaction (G × E) on all above measured traits (Table 2). Indeed1, dry weight (DW) was strongly influenced by region and genotype, with a highly significant G × E interaction (*P* < 0.001). Seed yield per plant also showed significant effects of region and genotype, as well as a significant G × E interaction, indicating differential genotype responses across environments. Similarly, TKW was significantly affected by region and genotype, with a significant G × E interaction, confirming environment‐dependent variation among genotypes.

**Figure 1:**
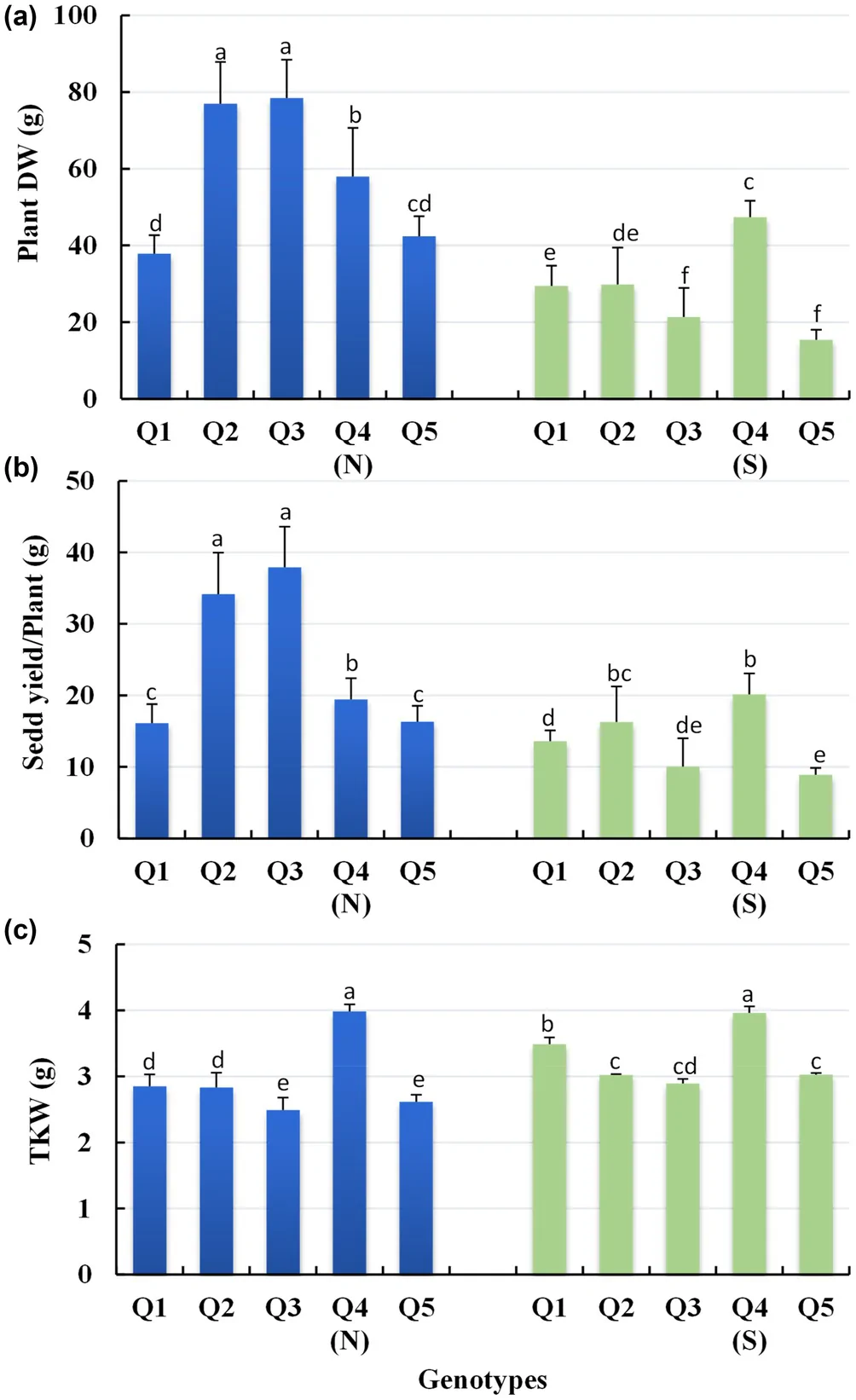
(a) Dry weight (DW), (b) seed yield and (c) thousand kernel weight (TKW) of five quinoa genotypes (Q1–Q5) grown at two different sites: North (N) and South (S). Data represent the means ± SE of 12 replicates. Bars sharing the same letter are not significantly different at *P* ≤ 0.05 according to Duncan's multiple range test.

**Table 2:** Two‐way ANOVA showing the effects of region (E), genotype (G) and their interaction (G × E) on agronomic and biochemical traits

Trait	Source of variation	*F* value	*P* value	Significance
DW	Region	130.3	1.93 × 10^−12^	***
	Genotype	21.5	1.87 × 10^−8^	***
	G × E	12.6	4.01 × 10^−6^	***
Seed yield/plant	Region	40.8	4.76 × 10^−7^	***
	Genotype	9.1	6.40 × 10^−5^	***
	G × E	10.9	1.41 × 10^−5^	***
TKW	Region	56.7	2.16 × 10^−8^	***
	Genotype	126.8	0.012	*
	G × E	7.7	2.16 × 10^−4^	***
Protein	Region	6.5	0.016	*
	Genotype	22.8	9.96 × 10^−9^	***
	G × E	87.3	4.44 × 10^−16^	***
Sugars	Region	11.5	0.002	**
	Genotype	0.6	0.655	NS
	G × E	4.5	0.005	**
Starch	Region	26.8	1.42 × 10^−5^	***
	Genotype	305.0	0.013	*
	G × E	194.7	0.026	*
Vitamin E	Region	7.4	0.011	*
	Genotype	34.4	8.21 × 10^−11^	***
	G × E	23.1	8.55 × 10^−9^	***
TPC	Region	199.3	8.66 × 10^−15^	***
	Genotype	142.7	0.011	*
	G × E	57.2	1.30 × 10^−13^	***
TFC	Region	980.2	0.013	*
	Genotype	770.0	0.006	**
	G × E	446.7	0.015	*
DPPH	Region	1.7	0.197	NS
	Genotype	40.2	1.22 × 10^−11^	***
	G × E	28.7	7.18 × 10^−10^	***
Saponin	Region	126.6	2.74 × 10^−12^	***
	Genotype	69.6	9.66 × 10^−15^	***
	G × E	29.4	5.30 × 10^−10^	***
Phytic acid	Region	0.2	0.625	NS
	Genotype	8.9	7.06 × 10^−5^	***
	G × E	2.4	0.075	NS
Tannins	Region	6.7	0.014	*
	Genotype	83.8	7.77 × 10^−16^	***
	G × E	42.1	6.84 × 10^−12^	***

NS, not significant.

* *P* < 0.05;

** *P <* 0.01;

*** *P* < 0.001.

### Nutritional properties

The nutritional properties of different quinoa seed genotypes, including protein, sugar, starch and vitamin E contents, were assessed under different cooking methods: Raw seeds (RS), Boiled seeds (BS) and Steamed seeds (SS) and obtained results are given in Figs 2 and 3.

**Figure 2:**
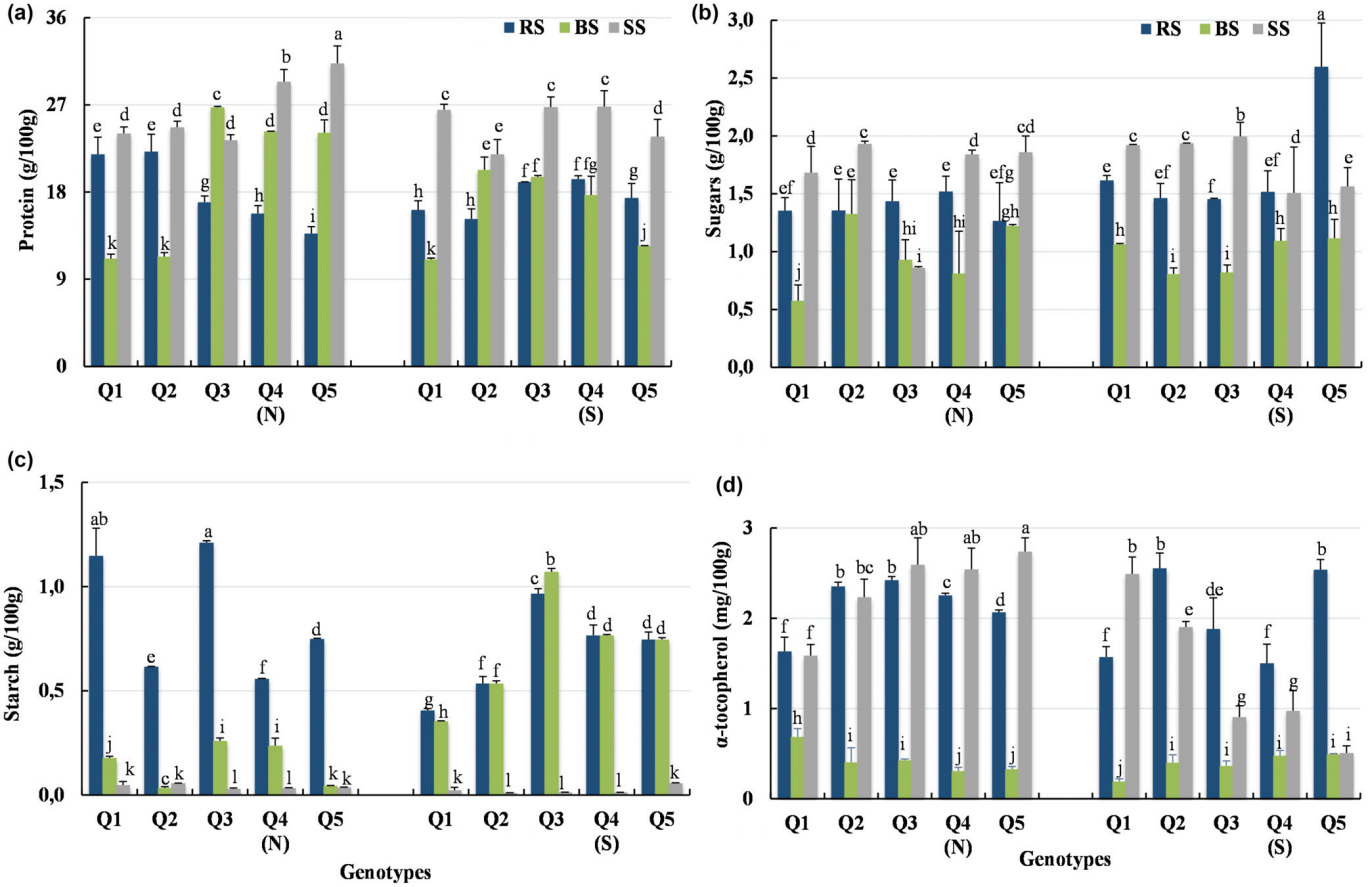
Seed nutritional traits: (a) protein; (b) soluble sugars, (c) starch and (d) vitamin E (α‐tocopherol) contents in five quinoa genotypes (Q1–Q5) grown at two sites, North (N) and South (S). BS, boiled seeds; RS, raw seeds; SS, steamed seeds. Data represent the mean ± SE of six replicates. Means followed by the same letter are not significantly different at *P* ≤ 0.05 according to Duncan's multiple range test (95%).

**Figure 3:**
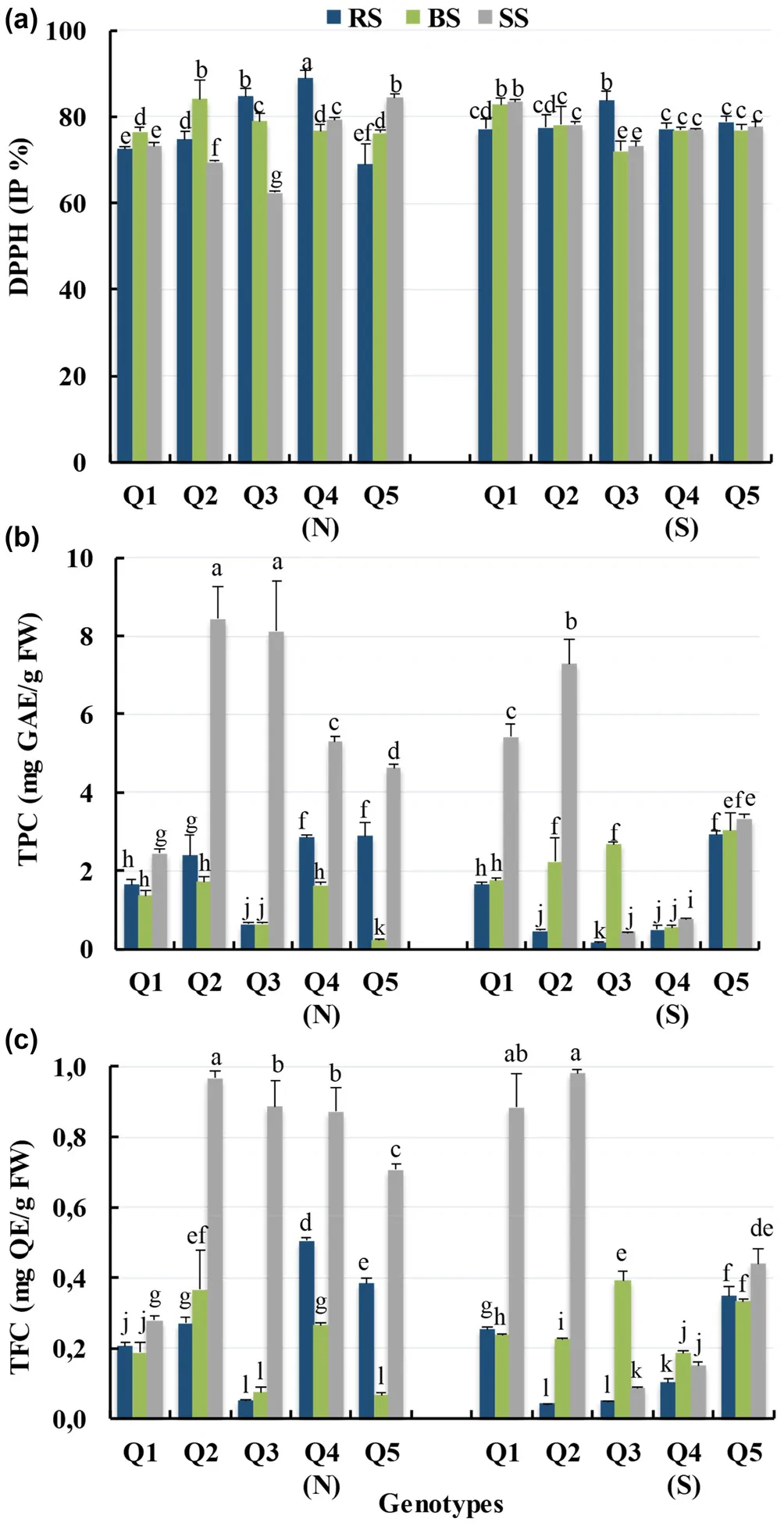
(a) Total phenolic content (TPC), (b) total flavonoid content (TFC) and (c) DPPH radical scavenging activity of five quinoa genotypes (Q1–Q5) grown at two sites, North (N) and South (S). BS, boiled seeds; RS, raw seeds; SS, steamed seeds. Data represent the mean ± SE of six replicates. Means followed by the same letter are not significantly different at *P* ≤ 0.05 according to Duncan's multiple range test (95%).

#### Protein content

Protein content in RS varied significantly among genotypes and between sites (Fig. 2(a)). The highest protein content was observed at the northern site in genotypes Q1 and Q2, whereas, at the southern site, it was highest in genotypes Q3 and Q4. In addition, results indicate genotypic variation in protein content across cooking methods and sites. Indeed, the boiling process, significantly, reduced protein content compared to RS, with a decrease of approximately 49% in genotypes Q1 and Q2 at the northern site and reductions of approximately 31% and 28% in genotypes Q1 and Q5, respectively, at the southern site (Fig. 2(a)). However, steaming significantly enhanced protein content in all genotypes, regardless of the seed origin. The highest protein content in SS was observed in genotypes Q4 and Q5 (~32%) from northern site and in genotypes Q1, Q3 and Q4 (~28%) from southern site. The most important increase in protein content following steaming were recorded in genotypes Q4 and Q5 from the northern site, with approximately 86% and 128% respectively, relative to RS. A similar effect was observed in genotypes from southern site with an increase of approximately 64% and 44% in genotypes Q1 and Q2, respectively.

#### Soluble sugars and starch content

Soluble sugar content in RS was generally similar among genotypes at both sites, except for genotype Q5, which exhibited a higher level at the southern site compared to the northern site (Fig. 2(b)). Boiling process (i.e. BS) resulted in a significant reduction in soluble sugars across most genotypes and sites. This decrease was more pronounced (57%) in genotype Q1 at the northern site and in genotype Q5 at the southern site. However, no significant change was observed in genotypes Q2 and Q5 at the northern site. The steaming process (i.e. SS) consistently led to higher soluble sugar content compared to both RS and BS. The highest sugar levels in SS were recorded in genotypes Q2 and Q5 at the northern site and in genotype Q3 at the southern site.

In RS, starch levels were generally higher at the northern site than at the southern site, with genotypes Q1, Q2 and Q3 exhibiting the highest values in the north. At the southern site, genotype Q3 showed the highest starch content, followed by Q4 and Q5 (Fig. 2(c)). Boiling (i.e. BS) induced a remarkable decline in starch content particularly in genotypes from northern sites. Similarly, SS showed a near‐complete loss of starch in most genotypes from both sites.

#### Vitamin E content

Vitamin E content (*α*‐tocopherol) varied significantly among quinoa genotypes, cooking methods, and sites (Fig. 2(d)). The data showed that, in RS, genotypes Q2 and Q5 from the southern site exhibited the highest level of vitamine E content (2.5 mg per 100 g). Boiling induced a significant decrease vitamin E content in all genotypes from both sites. The decrease ranged from 80% to 87% for most genotypes, except for Q1 from the northern site and Q4 from the southern site, which showed decreases of approximately 58% and 68%, respectively. By contrast, steaming had no significant effect on vitamin E content in any genotype from the northern site. Moreover, an increase of approximately 32.4% and 58.5% was observed in Q5 from northern site and Q1 from southern site, respectively.

### Functional and antinutritional constituents

#### DPPH radical scavenging capacity

Our results demonstrated that the DPPH assay revealed a high antioxidant capacity in RS, particularly for genotype Q3 (~84%) from both sites and for Q4 (~88%) from the southern site (Fig. 3(a)). Genotype Q1 from the southern site showed the highest percentage inhibition of the DPPH (IP%) in both BS and SS as compared to other genotypes. However, at the northern site, genotypes Q2 and Q5 showed the highest IP in BS and RS, respectively. Interestingly, we noted a stimulation in antioxidant capacity of approximately 22.4% in SS of Q5 and approximately 12.4% in BS of Q2 from northern site, relative to their corresponding RS.

#### Total phenolic and flavonoid contents (TPC and TFC)

The variation in total phenolic and flavonoid content among genotypes, sites and cooking methods is shown in Fig. 3(b), (c). The highest levels of TPC and TFC in RS were observed in genotypes Q4 and Q5 at the northern site, and in genotypes Q1 and Q4 at the southern site. Across all genotypes and both sites, SS consistently exhibited the highest polyphenol levels, followed by RS, whereas BS showed the lowest values. In fact, boiling induced a significant decrease in TPC and TFC contents, particularly in genotypes Q4 and Q5 from the northern site. Specifically, TPC decreased by approximately 43% and 91%, and TFC by approximately 47% and 82% in Q4 and Q5, respectively, compared to their respective RS.

Interestingly, steaming resulted in a significant increase in both polyphenol and flavonoid contents compared to RS across all genotypes and at both the northern and southern sites. The magnitude of this increase varied among genotypes and compounds. At the northern site, increases in TPC and TFC of SS were observed in genotypes Q2 and Q4, with TPC increases of approximately 254% and 86%, respectively, and corresponding TFC increases of approximately 256% and 73%, relative to their RS. This increase was more pronounced in genotype Q3, which exhibited a more than ten‐fold enhancement in both TPC and TFC compared to RS. Similarly, at the southern site, genotypes Q1 and Q2 exhibited the highest increases in TPC and TFC. In genotype Q1, TPC and TFC increased by approximately 230% and 249%, respectively. Notably, SS of genotype Q2 from the southern site showed an extremely pronounced increase, with more than a 15‐fold enhancement in both TPC and TFC compared to RS.

#### Saponin levels

Saponin content in RS showed significant genotypic variation at both the North (N) and South (S) sites (Fig. 4(a) and Table 2). At the northern site, saponin levels in RS ranged approximately from 0.20 to 1.50%. The highest value was observed in Q5 (1.52%), followed by Q1 and Q2 (0.7%). Intermediate values were recorded for Q4 (0.6%), wheeras the lowest saponin content was found in Q3 (0.2%). At the southern site, saponin contents in R) were generally lower, ranging from approximately 0.10 to 0.7%. Genotype Q1 exhibited the highest saponin level (0.73%), followed by Q5 (0.4%) and Q4 (0.2%). The lowest values were observed in Q2 (0.1%) and Q3 (approximately 0.10%).

**Figure 4:**
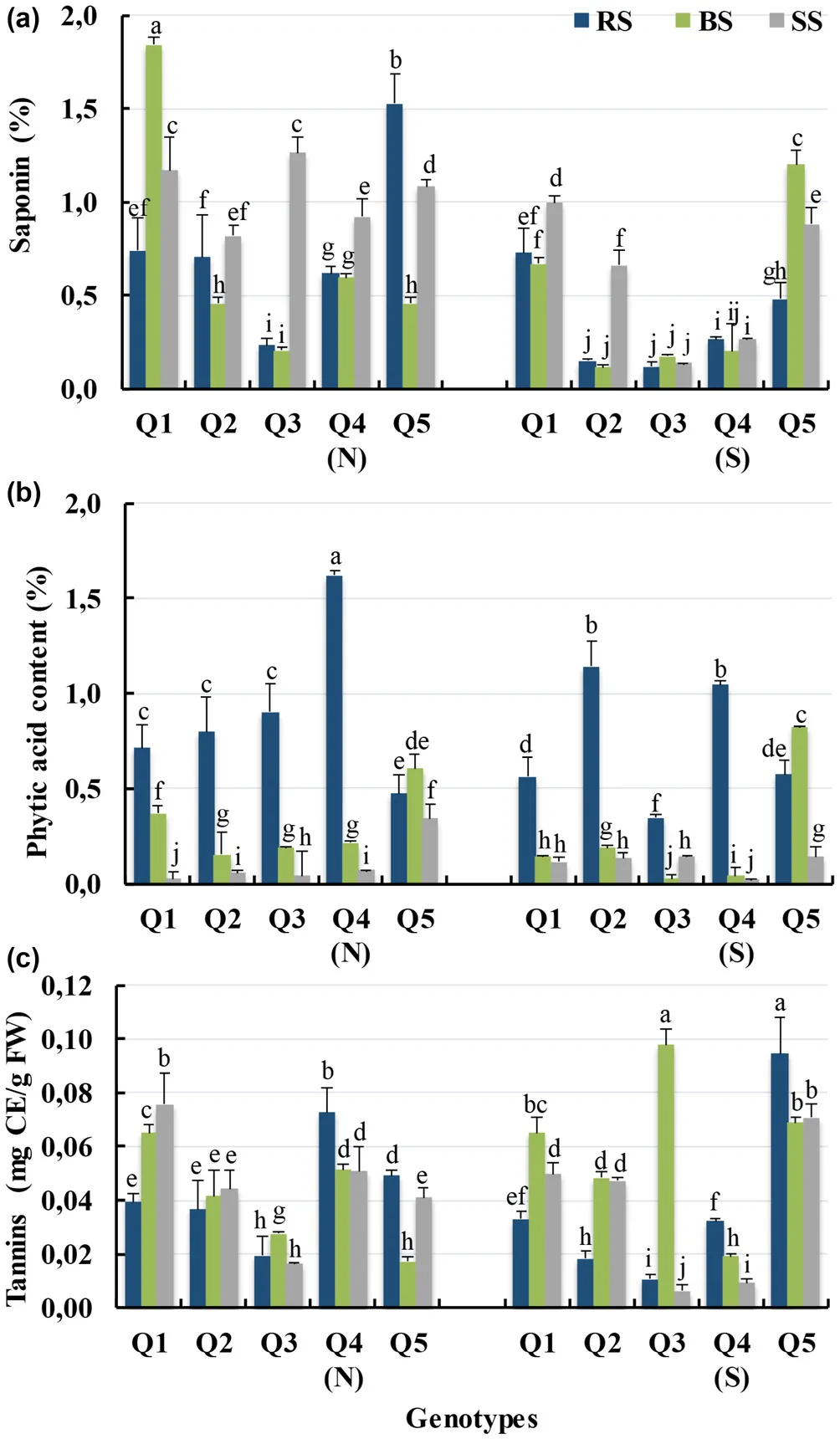
Seed antinutritional factors: (a) saponin, (b) phytic acid and (c) tannin contents of five quinoa genotypes (Q1–Q5) grown at two sites, North (N) and South (S). BS, boiled seeds; RS, raw seeds; SS, steamed seeds. Data represent the mean ± SE of six replicates. Means followed by the same letter are not significantly different at *P* ≤ 0.05 according to Duncan's multiple range test (95%).

Boiling significantly affected saponin content across all genotypes at both sites. Boiling significantly increased saponin content, particularly in Q1 from the northern site and Q5 from the southern site, with approximately 148% and 150%, respectively, as compared to RS. By contrast, Q5 from southern site, exhibited a significant reduction in saponin content of approximately 70%.

SS exhibited higher saponin levels than RS, although the magnitude of the increase varied markedly among genotypes and between sites. For genotypes from the northern site, steaming resulted in increases in saponin content of approximately 58%, 17% and 48% in Q1, Q2 and Q4, respectively, compared to RS. Similarly, SS of genotypes from the southern site showed higher saponin levels than RS; however, the increases were generally smaller than those observed at the northern site. Steaming increased saponin content by approximately 37% and 83% in Q1 and Q5, respectively. Particularly, the increase in saponin content was most pronounced in genotype Q3 from the northern site and genotype Q2 from the southern site, which exhibited more than a three‐fold increase compared to RS.

#### Phytic acid content

Our results showed that phytic acid content was more accumulated in RS of genotypes from both sites compared to BS and SS, with the highest levels observed in genotype Q4 at both sites (Fig. 4(b)). Both boiling and steaming processes induced a reduction in phytic acid content, with the decrease being more pronounced following steaming, particularly in genotype Q4 from both sites. For genotypes from the northern site, phytic acid reduction ranged from 92% to 96% in SS, and from approximately 48%, 80%, 78% and 86% in BS for genotypes Q1, Q2, Q3 and Q4, respectively. BS of genotype Q5 from the northern site showed no significant change, and this genotype also exhibited the lowest decrease (27%) after steaming compared to RS. Two‐way ANOVA revealed no significant regional effect on phytic acid content (*P* = 0.625) (Table 2), whereas a highly significant genotype (G) effect was observed (*P* < 0.001). Statistical analysis also indicated a highly significant genotype × cooking process interaction (*P* < 0.001) for this parameter (Table 3).

**Table 3:** Summary of two‐way ANOVA showing the effects of genotype (G), cooking method (C), and their interaction (G × C) on nutritional and antinutritional traits

	Source of variation	*F* value	*P* value	Significance
Protein	G	6.32	1.35 × 10^−4^	***
	C	85.13	0.001	***
	Interaction G × C	7.44	8.10 × 10^−8^	***
Sugars	G	2.53	0.045	*
	C	81.20	0.006	**
	Interaction G × C	1.81	0.083	NS
Starch	G	13.45	6.99 × 10^−9^	***
	C	534.71	0.0323	*
	Interaction G × C	8.77	4.09 × 10^−9^	***
Saponin	G	19.07	8.18 × 10^−12^	***
	C	9.15	2.16 × 10^−4^	***
	Interaction G × C	1.15	0.339	NS
Vit. E	G	1.36	0.252	NS
	C	120.61	0.042	*
	Interaction G × C	1.81	0.083	NS
TPC	G	4.87	0.001	**
	C	28.33	1.45 × 10^−10^	***
	Interaction G × C	4.95	3.23 × 10^−5^	***
TFC	G	3.59	0.009	**
	C	26.71	4.19 × 10^−10^	***
	Interaction G × C	3.95	4.07 × 10^−4^	***
DPPH	G	2.33	0.06	NS
	C	5.39	0.01	*
	Interaction G × C	12.03	4.64 × 10^−12^	***
Phyt. A.	G	4.82	0.0013	**
	C	157.37	0.022	*
	Interaction G × C	13.26	4.30 × 10^−13^	***
Tannins	G	9.39	1.59 × 10^−6^	***
	C	5.06	0.008	**
	Interaction G × C	7.43	8.41 × 10^−8^	***

NS, not significant.

* *P* < 0.05;

** *P* < 0.01;

*** *P* < 0.001.

#### Total tannin content

Figure 4(c) shows the total tannin content for quinoa genotypes from both sites in RS, BS and SS. Genotypes Q4 and Q5 exhibited the highest levels of tannins in RS, whereas genotype Q3 had the lowest content at both sites. Tannin concentration was affected differently by both the cooking method and the origin of the seeds. Boiling induced an increase in tannin content for genotypes Q1, Q2 and Q3 from both sites, compared to RS, whereas it led to a decrease for Q4 and Q5. Specifically, for BS from the northern site, tannin reduction was approximately 29% and 65% for Q4 and Q5, respectively, and approximately 41% and 27% for seeds from the southern site. Regarding SS, tannin content increased particularly in genotypes Q1 and Q2, whereas it decreased for Q3, Q4 and Q5 from both sites. This reduction was more pronounced in SS from the southern site, with reductions of approximately 40%, 71% and 25% for Q3, Q4 and Q5, respectively.

## DISCUSSION

As a superfood, quinoa is generally consumed after cooking, with boiling and steaming representing the two most common household cooking methods.^38^, ^39^ Both seed origin and the cooking process can influence the composition of quinoa seeds, particularly affecting heat‐sensitive compounds and water‐soluble molecules.^33^ In this context, the present study aimed to investigate the impact of boiling and steaming on the nutritional and antinutritional qualities of five different quinoa genotypes harvested from two contrasting regions in Tunisia. The first site, located in the south, is one of the driest regions with high soil salinity, whereas the second site, located in the north, is considered a humid region. Indeed, understanding the relative contributions of environmental and genetic factors to trait expression is essential for improving crop performance and stability across diverse growing conditions. The two‐way ANOVA (Table 2) analysis clearly demonstrates that environmental conditions, genotype, and their interaction jointly regulate key agronomic and nutritional traits. Both region and genotype exerted highly significant effects (*P* < 0.001) on the evaluated traits (DW, seed yield and TKW), indicating that environmental conditions play a dominant role in biomass accumulation and yield production, at the same time as revealing significant genetic variability among the tested genotypes. In this study, the southern site was characterized by arid conditions and soil salinity (see Supporting information, Table S1), which strongly contributed to the reduced growth and yield performance observed in most genotypes compared to the northern site, where the highest biomass and yield values were recorded (Fig. 1). These findings are consistent with previous studies^40^, ^41^ showing that resource availability, climate and soil characteristics strongly influence the growth and productivity of quinoa genotypes, whereas genetic background determines the efficiency with which plants exploit these conditions. The significant G × E interaction further reveals that genotype performance was environment‐dependent, with regional differences being particularly pronounced for genotypes such as Q1 and Q2. This differential response demonstrates genotypic sensitivity to environmental conditions influencing biomass production and seed development, underlining the necessity of multi‐environment evaluation for the identification of high‐yielding and stable genotypes.

Regarding G × E effects on nutritional and anti‐nutritional traits in RS, the two‐way ANOVA revealed that region (R), genotype (G) and their interaction (R × G) significantly influenced most traits (Table 2), highlighting the complex interplay between environmental conditions and genetic background in determining grain quality. The PCA of RS explained 53% of the total variation with the first two principal components (PC1 = 32%, PC2 = 21%) (Fig. 5(a), (b)). PC1 separated genotypes primarily based on protein, saponins, total phenolics (TPC), total flavonoids (TFC) and tannins, with genotypes Q1 and Q5 associated with higher levels of these compounds, suggesting their superior nutritional quality and bioactive potential in the unprocessed state (RS). By contrast, Q3 and Q4 were associated with higher starch content, phytic acid, and DPPH antioxidant activity, reflecting an alternative nutrient profile. Genotype Q2 displayed intermediate characteristics, occasionally overlapping with both clusters, indicating moderate levels of protein‐ and starch‐related traits. Regional differences were evident along PC2, with North and South samples showing slight separation, indicating environmental influences on nutrient composition even before processing. These observations are in agreement with previous studies showing that genotype and the growing environment jointly influence the nutritional quality of quinoa. For example, Pedrali *et al*.^42^ reported that protein, saponins, and antioxidant compounds vary significantly among genotypes and cultivation locations. Similarly, Bhargava *et al*.^43^ observed that starch and anti‐nutritional factors, such as phytic acid, are strongly affected by both genotype and environmental conditions, supporting the patterns observed in this study. Consequently, this baseline is essential for understanding how boiling and steaming alter the nutritional and anti‐nutritional profiles of seeds.

**Figure 5:**
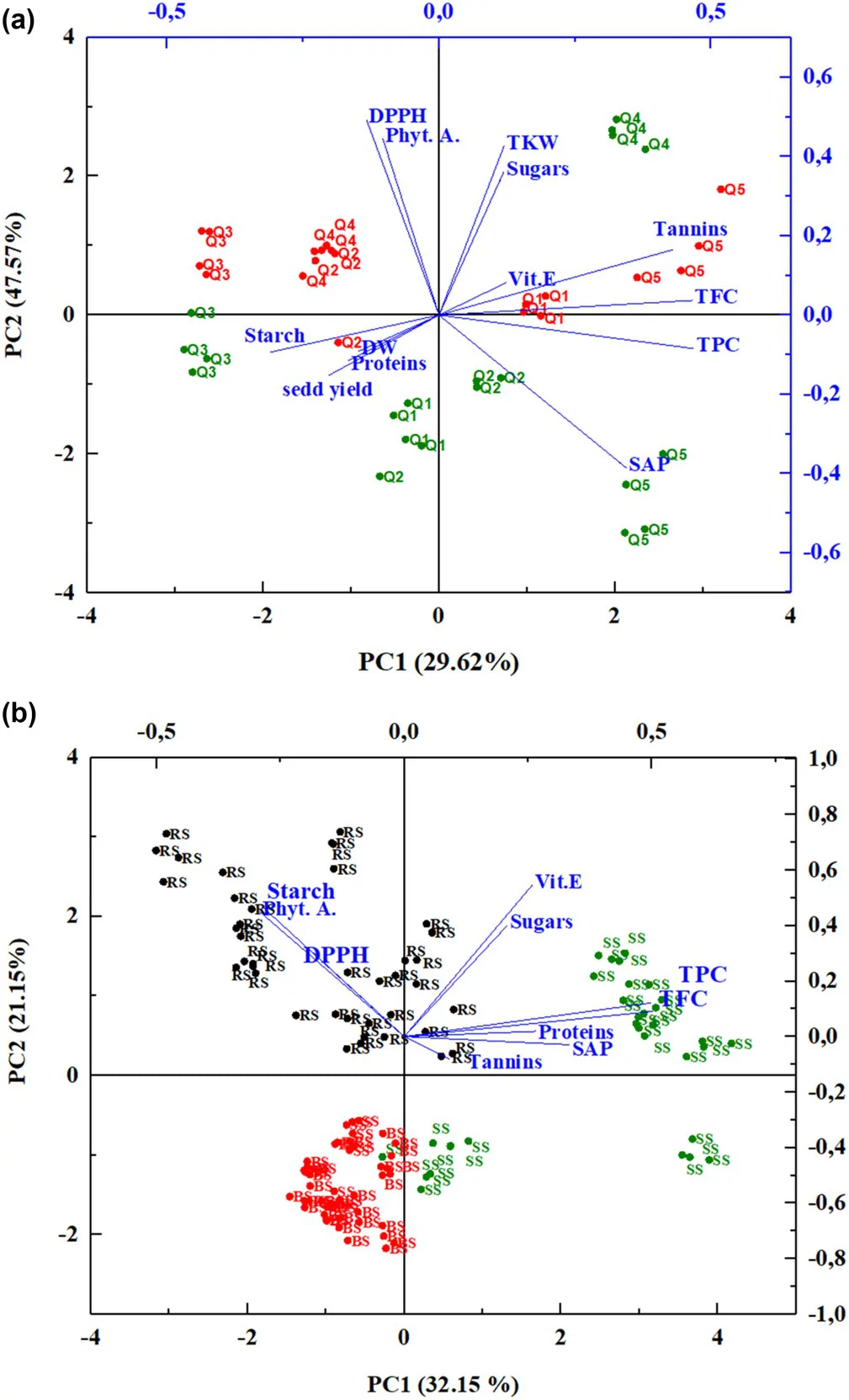
Principal component analysis (PCA) biplots showing: (a) the distribution of nutritional and antinutritional traits in relation to genotypes and region (genotypes from the northern site shown in green and those from the southern site shown in red) and (b) the distribution of nutritional and antinutritional traits in relation to cooking methods (BS, boiled seeds; RS, raw seeds; SS, steamed seeds). Each point represents an individual replicate, and clustering indicates similarities among treatments in response to the measured variables.

Food processing via thermal treatments transforms RS or foods into edible forms, often increasing the bioavailability of nutrients by inactivating anti‐nutritional factors. Among thermal methods, boiling (i.e. BS) and steaming (i.e. SS) are the most commonly applied, and both significantly affect the nutritional and anti‐nutritional composition of seeds.^44^, ^45^, ^46^ Understanding these effects is essential for optimizing processing techniques to retain nutritional quality at the same time as minimizing anti‐nutritional factors. Heat treatments can alter the levels of proteins, carbohydrates, vitamins and bioactive compounds while reducing anti‐nutrients such as phytic acid, tannins and saponins.^47^ The magnitude of these changes depends not only on the processing method and duration, but also on the genotype because different genetic backgrounds vary in their nutrient content and sensitivity to heat. A recent study by Dostalíková *et al*.^38^ demonstrated that boiling, involving direct contact with water, often causes greater leaching of water‐soluble nutrients, whereas steaming generally preserves more bioactive compounds. This study also demonstrated that steamed quinoa seeds exhibited higher total phenolic content and greater retention of bioactive compounds compared to BS.^38^

In our study, protein content remained relatively stable during both cooking methods, with steaming even showing an increase in protein levels in genotypes Q1, Q2 and Q3. This suggests that steaming may better preserve protein compared to boiling, leading to higher measured protein content, although it does not generate new protein. The apparent increase in protein during cooking is likely a result of the reduction in soluble compounds, such as sugars and ions, which decreases global seed mass at the same time as leaving protein largely intact, thereby increasing its relative proportion.^48^ The stability of protein during cooking can also be attributed to the presence of numerous disulfide bonds, which help quinoa proteins maintain their structural integrity and retain their nutritional and functional properties.^49^ However, protein quality is not only determined by total protein content, but also by the preservation of essential amino acids, particularly lysine, which is highly sensitive to processing conditions.^50^ Steaming may be a more favorable cooking method than boiling because it better preserves amino acids and minimizes nutrient losses, whereas prolonged boiling at high temperatures can promote chemical interactions between proteins and reducing sugars (Maillard reactions), potentially decreasing the bioavailability of essential amino acids.^51^ Boiling significantly reduced protein levels, particularly in genotypes Q1 and Q2 from the northern site, with moderate reductions observed in genotypes from the southern site. This reduction can be attributed to the leaching of water‐soluble proteins and partial denaturation as a result of heat exposure and prolonged contact with water during boiling. By contrast, the increase in protein content observed during steaming in genotypes Q4 and Q5 from the northern site can be explained by minimal leaching, which preserves protein, coupled with a relative increase in protein proportion because of the loss of water and soluble carbohydrates. Consistent with this, our study showed that the highest retention of soluble sugars during steaming occurred in genotypes Q2 and Q5 at the northern site and Q3 at the southern site, indicating that steaming is more effective than boiling at maintaining water‐soluble carbohydrates, whereas boiling primarily reduces sugars through leaching into the cooking water. Starch content was also strongly affected by thermal processing, declining significantly during both boiling and steaming, particularly in genotypes from the northern site. This reduction is likely a result of heat‐induced gelatinization and starch leakage, where swelling of starch granules during cooking disrupts their structure, allowing some starch molecules to diffuse into the cooking medium.^52^ Although steaming reduces direct water contact and thus minimizes sugar and protein leaching, it still causes partial starch gelatinization and leakage, explaining the decrease in starch content observed across genotypes.

Regarding secondary metabolites, our results (Figs 3, 4, 5, 6 and Table 3) indicate that variations in total flavonoids, phenolic compounds, tannins, saponins and antioxidant capacity are strongly influenced by genetic factors, cooking processes, and environmental conditions, highlighting their combined role in shaping the bioactive profile of quinoa seeds. RS exhibited high DPPH radical scavenging capacity, particularly in genotypes Q3 and Q4, reflecting their naturally high antioxidant potential. However, thermal processing altered this response depending on the method applied. Boiling (i.e. BS) tended to reduce antioxidant capacity in most genotypes, which can be attributed to thermal degradation of antioxidants. By contrast, steaming (i.e. SS) either preserved or enhanced antioxidant activity in several genotypes, as evidenced by the notable stimulation of DPPH activity in SS of Q5 and BS of Q2 at the northern site. This enhancement may result from the heat‐induced liberation of bound phenolic compounds or structural modifications that improve extractability and radical scavenging efficiency. Because total phenolic content (TPC) and total flavonoid content (TFC) are key contributors to antioxidant activity, steaming consistently resulted in higher TPC and TFC across genotypes and sites compared to both RS and BS, whereas boiling caused significant losses. The pronounced reduction in TPC and TFC following boiling, particularly in genotypes Q4 and Q5 from the northern site, could be associated with the combined effects of thermal degradation and leaching of water‐soluble phenolics during prolonged exposure to hot water. Interestingly, the marked enhancement of phenolic compounds following steaming, reaching several‐fold increases in some genotypes, suggests that steaming promotes the release of phenolic compounds bound to cell wall components or macromolecules. Heat‐induced softening of the seed matrix and partial disruption of cell structures could facilitate the accessibility and extractability of phenolics and flavonoids, leading to higher measured concentrations, as reported by Frlin *et al*.,^53^ who demonstrated that heat treatments enhance phenolic extractability by disrupting structural matrices.

**Figure 6:**
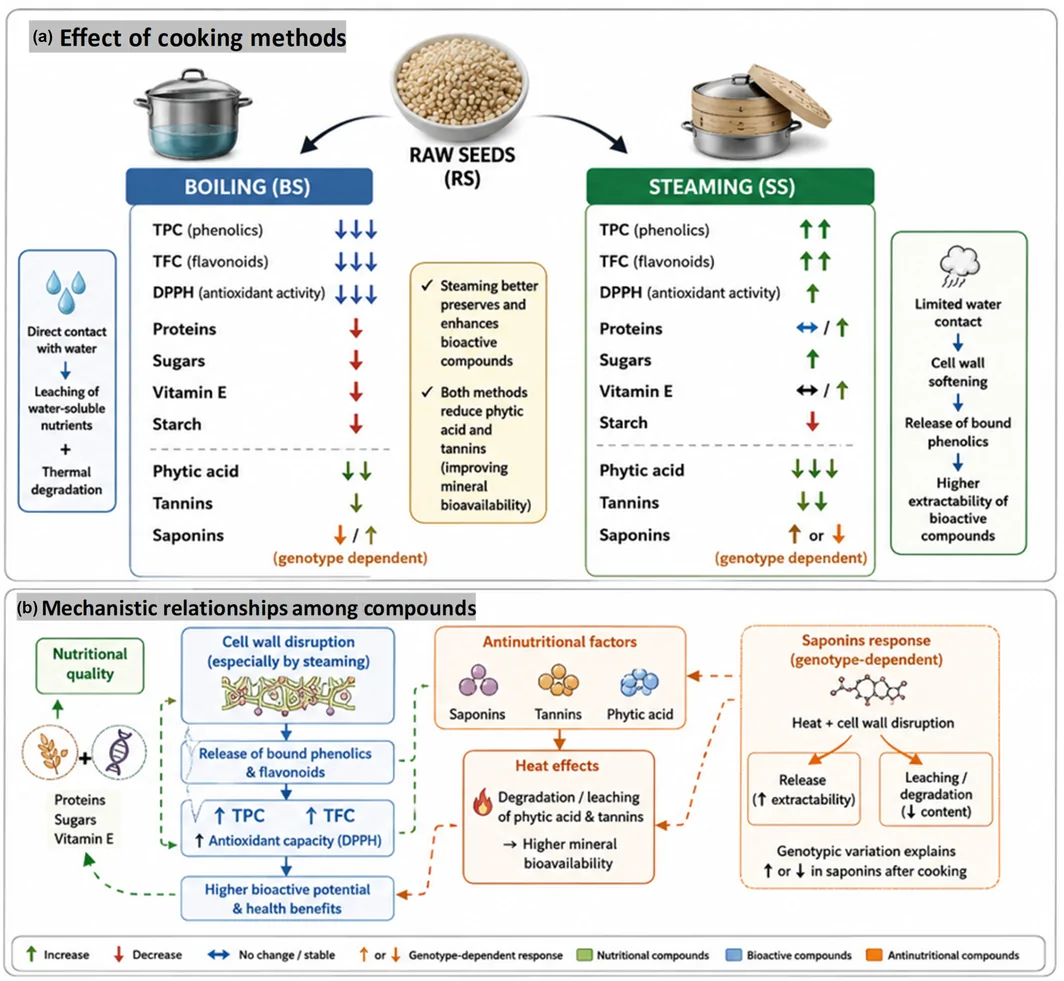
Integrated conceptual model illustrating the combined effects of genotype, growing environment, and cooking methods on the nutritional, bioactive and antinutritional composition of quinoa seeds. (A) Thermal processing differentially modifies seed composition depending on the cooking method. Boiling promotes nutrient leaching and thermal degradation because of direct contact with water, leading to greater losses of water‐soluble compounds and antioxidant constituents. By contrast, steaming minimizes leaching, enhances the extractability of bound phenolic compounds through cell wall softening, and generally improves the retention of proteins and antioxidant compounds at the same time as reducing phytic acid and tannins. The response of saponins is genotype‐dependent as a result of differences in their localization, extractability and thermal stability. (B) Proposed mechanistic relationships among the measured traits. Steaming‐induced disruption of seed cellular structures facilitates the release of bound phenolics and flavonoids, increasing TPC, TFC and antioxidant capacity (DPPH). Heat treatment also promotes the degradation of phytic acid and tannins, potentially improving mineral bioavailability, whereas changes in saponin content result from the balance between enhanced extractability and leaching or thermal degradation.

The levels of key antinutritional compounds, saponins, phytic acid and tannins, are also affected by the cooking process, with boiling and steaming producing distinct changes depending on genotype and growing environment (Figs 4 and 6 and Table 3). In RS, significant genotypic variation was observed, with northern‐site genotypes generally exhibiting higher saponin content than southern‐site genotypes. The apparent increase in saponins following steaming or boiling (as observed in genotype Q1 from the northern site and Q5 from the southern site) could result from heat‐induced breakdown of seed cell walls, which releases saponins previously bound within the seed matrix, increasing their extractability. Our observations are consistent with previous studies showing that thermal processing can increase saponin content, such as in sweet potato leaves after steaming^54^ and in quinoa following pressure cooking.^55^ Conversely, the reduction in saponin content observed in some genotypes in our study may be attributed to thermal degradation or the leaching of water‐soluble saponins into the cooking water. Quinoa saponins are triterpenoid glycosides that dissolve in water during prolonged boiling, resulting in lower residual levels in the cooked seeds.^56^ This genotypic variability likely reflects differences in saponin localization within the seed, their association with cell wall components and the inherent structural stability of these glycosides.

Both phytic acid and tannin contents in quinoa seeds were strongly influenced by cooking methods, with genotype‐specific responses. In agreement with our findings, Devi *et al*.^57^ reported that tannin content in quinoa and buckwheat seeds decreased significantly by 20% and 83%, respectively, following germination. They also demonstrated that thermal processing effectively reduces several antinutritional factors, including phytic acid, tannins, trypsin inhibitors, saponins and oxalates. Similarly, in the present study, phytic acid content was highest in RS and decreased significantly after both boiling and steaming, with steaming resulting in a greater reduction, particularly in genotype Q4. This decrease is expected because of heat‐induced degradation of phytic acid into soluble compounds such as inositol and phosphates, which enhances mineral bioavailability. These findings are consistent with previous reports on cooked pseudocereals, including quinoa and amaranth, where boiling and other heat treatments reduce antinutrients and improve the bioaccessibility of minerals such as iron and zinc.^58^ These variations indicate that the impact of heat on antinutritional compounds depends on their localization, solubility and interactions with seed structures.

## CONCLUSIONS

This study demonstrates that both pre‐harvest factors (genotype and growing environment) and post‐harvest processing methods (boiling and steaming) play critical and complementary roles in shaping the nutritional and antinutritional profiles of quinoa seeds (Fig. 6). The significant effects of region, genotype and their interaction highlight the strong influence of environmental conditions on agronomic performance and baseline grain quality, at the same time as confirming the importance of genetic diversity for nutrient composition and processing response. Overall, Q3 exhibited the best nutritional profile across both northern and southern regions, combining high protein, starch, vitamin E, antioxidant capacity and the lowest levels of antinutritional compounds (saponins, tannins and phytic acid), making it the most promising genotype for nutritional quality and broad adaptation. In contrast, Q5 showed the least favorable profile, characterized by low yield performance, high saponin and tannin contents, and less consistent nutritional improvements after processing. Thermal processing induced marked biochemical changes, with boiling generally promoting nutrient losses through leaching and degradation, particularly of soluble sugars and phenolic compounds, whereas steaming better preserved protein content, enhanced phenolic and flavonoid extractability, maintained antioxidant activity, and effectively reduced phytic acid and tannin contents. Collectively, these findings identify Q3 as the most suitable genotype for cultivation in both northern and southern Tunisia, providing farmers with a genotype that combines superior agronomic performance and nutritional quality after cooking. Future research should validate these findings across a wider range of agroecological environments and growing seasons to confirm the stability of genotype performance. Further studies should also assess consumer acceptance, sensory quality, shelf life and industrial‐scale processing to facilitate the development of value‐added quinoa‐based products.

## FUNDING INFORMATION

No funding was received for this study.

## CONFLICTS OF INTEREST

The authors declare that they have no conflicts of interest.

## AUTHOR CONTRIBUTIONS

All of the authors have made a significant contribution to this paper. RG was responsible for writing the original draft. RG and AM were responsible for validation. RG, RBY, HG and AM were responsible for methodology. RG and AM were responsible for investigations. RG, RBY, HG, HBJ and AM were responsible for formal analysis. RG was responsible for data curation. HBJ and AM were responsible for reviewing and editing. AM was responsible for supervision, visualization and conceptualization. All authors read and approved the final version of the manuscript submitted for publication.

## Supporting information


**Table S1.** Geographic distribution, climatic conditions (average 2022–2024) and soil characteristics (depth 0–25 cm) of the two‐study sites in south and north of Tunisia (source: Climate-Data.org). The physical and mineralogical properties of soils were measured according to the methods described by Blake & Hartge (1986)^59^.

## Data Availability

The data that support the findings of this study are available from the corresponding author upon reasonable request.
