# Supplementary material for: Interactive effects of cooking method, genotype and environment on nutritional and antinutritional quality of quinoa (Chenopodium quinoa Willd.) seeds
Source: J Sci Food Agric. 2026 Aug 28;107(1):560–73. doi: 10.1002/jsfa.71022 (PMC13756580; doi:10.1002/jsfa.71022)
Supplement: Supplementary file 1 [file JSFA-107-560-s001.docx]

**Table 1S.** Geographic distribution. climatic conditions (average 2022-2024) and soil characteristics (depth 0-25 cm) of the two-study sites in south and north of Tunisia (Source: Climate-Data.org). The physical and mineralogical properties of soils were measured according to the methods described by Blake & Hartge (1986)

|  | **Northern site (N)** | **Southern site (S)** |
| --- | --- | --- |
| **Geographic distribution and climatic conditions** | | |
| GIS coordinates | 36°37'14.0"N 10°32'42.4"E | 33°30'25.2"N 8°50'11.2"E |
| Climate region | Humid | Oasis (Sahara) |
| Average rainfall (mm) * | 410 | 7.1 |
| Average temperature (°C) * | 18.3 | 21.7 |
| Max. temperature * | 26.7 | 27.8 |
| Min. temperature * | 10.9 | 14.9 |
| Relative humidity (%) | 72.9 | 43.7 |
| **Soil characteristics and Nutrients availability** | | |
| Clay (%) | 22.2 | 8.20 |
| Silt (%) | 7.08 | 1.90 |
| Sand (%) | 70.72 | 89.9 |
| CaCO_3_ (%) active | 6.09 | <3 |
| CaCO_3_ (%) total | <3 | <4 |
| pH | 8.1 | 8.76 |
| OM (%) | 1.73 | 0.24 |
| CE1:5 (mS/cm) | 0.2 | 5.78 |
| N total (%) | 1.17 | 0.29 |
| C/N | 8.6 | 9.65 |
| P_2_O_5_ assimilable (mg.kg^-1^) | 118.2 | 18.35 |
| K_2_O (mg.kg^-1^) | 596.6 | 470.8 |
| Ca (mg.kg^-1^) | 4221.1 | 23379.2 |
| Mg (mg.kg^-1^) | 284.1 | 518.6 |
| Na (mg.kg^-1^) | 42.6 | 1108.6 |

*Abbreviations*: OM. organic matter. Max. maximum. Min. minimum. Avg. average. EC. electrical conductivity. N. north. E. east. * represent the year average (2022 and 2024).
